# Contribution of calcium in drinking water from a South American country to dietary calcium intake

**DOI:** 10.1186/s13104-020-05308-7

**Published:** 2020-10-02

**Authors:** Gabriela Cormick, Mercedes Lombarte, Nicole Minckas, Andrés Porta, Alfredo Rigalli, Jose M. Belizán, Natalia Matamoros, Maela Lupo

**Affiliations:** 1 Department of Mother and Child Health Research, Institute for Clinical Effectiveness and Health Policy (IECS-CONICET), Emilio Ravignani 2024, Buenos Aires, Argentina; 2 Departamento de Salud, Universidad Nacional de La Matanza (UNLAM), 1754 San Justo, Argentina; 3 Bone Biology Laboratory, School of Medicine, Rosario National University, 2000 Rosario, Santa Fe Argentina; 4 Centro de Investigaciones del Medio Ambiente CIM, UNLP-CONICET, Calle 47 esquina 115, La Plata, Argentina; 5 Instituto de Desarrollo E Investigaciones Pediátricas “Prof. Dr. Fernando E. Viteri” Hospital de Niños “Sor María Ludovica de La Plata (IDIP), Ministerio de Salud/Comisión de Investigaciones Científicas de La Provincia de Buenos Aires, La Plata, Argentina

**Keywords:** Bottled water, Calcium intake, Drinking water

## Abstract

**Objective:**

To describe the calcium concentration of tap and bottled waters from Argentina and to estimate the contribution of drinking water to calcium recommendations.

**Results:**

Calcium concentrations provided by water authorities ranged from 6 to 105 mg/L. The mean calcium level of samples analysed at the Laboratorio de Ingeniería Sanitaria, National University of La Plata was 15.8 (SD ± 13.2) mg/L and at the Bone Biology Laboratory of the National University of Rosario was 13.1 (± 10.0) mg/L. Calcium values of samples from supply systems and private wells was similar. Most bottled waters had calcium levels well below 50 mg/L. The intake of one litre of drinking water from Argentina could represent in average between 1.2 and 8.0% of the calcium daily values for an adult.

## Introduction

The amount of calcium in drinking water is variable, it can range from negligible levels to values higher than those contained in several dairy products, this variability depends on the origin, treatment received and distribution system [1, 2]. Data from Canada and the United States of America show that the average calcium concentration in tap water varies between 6.8 to 135 mg/L [3]. Also, a study conducted in Spain registered values of calcium in tap water ranging from 0.5 to more than 200 mg/L [4]. Higher values of 64 to 523 mg/L were published in another study conducted in Algeria [5]. The amount of ionic calcium in commercially bottled water is also variable and levels of calcium can reach more than 400 mg/L [6].

Calcium in water is basically found in its ionic form (soluble calcium), which enhances absorption in the gastrointestinal tract [7]. Pooled data from 4 studies shows a mean absorbability ratio for calcium in water and in milk of 1.084 ± 0.043 [2]. With this high bioavailability, water consumption could improve daily calcium intake and contribute to meet daily recommendations [8].

An adequate calcium intake is important for the prevention of hypertension, preeclampsia and for bone health maintenance [9, 10]. It has also been associated with the reduction of renal stones, increased body mass index, insulin resistance and colorectal cancer [11–15].

Calcium intake is inadequate in several regions of the world. In spite of the well-known benefits of calcium intake on the prevention of preeclampsia, pregnant women in LMICs show values well below recommendation [20–23]. In Argentina, the 2005 National Nutrition and Health Survey (ENNyS) showed that more than 94% of women and about half of children age 2 to 5 years do not reach values of adequate intake [24, 25]. However, the survey did not assess water intake so calcium intake could be higher than that reported.

Data published on the mineral content of water in Argentina is scarce, as quality control does not require analysis of calcium [26]. Some food composition tables only have commercially bottled water information while others do not report any type of water [27, 28]. The objective of this article is to describe the level of calcium of tap water samples supplied by municipal pipelines of different localities of Argentina as well as of bottled waters commercially available in the country. The secondary objective is to estimate the contribution of drinking water from Argentina to calcium intake recommendations.

## Main text

### Methods

We contacted provincial water authorities of Argentina to obtain data on the calcium content of tap water and reported the values provided by each authority. In addition, we obtained information from tap water samples from different municipalities of the country remitted to the Bone Biology Laboratory of the National University of Rosario (UNR) and the Laboratorio de Ingeniería Sanitaria, National University of La Plata (UNLP). All samples were obtained from drinking tap water that was either from private wells or from water supply systems. Water from private wells was defined as groundwater of different depths pumped into a private dwelling. Water supply systems were defined as water provided by local authorities, obtained from groundwater or superficial water that received different treatment procedures before distribution.

The calcium concentration of water samples remitted to laboratories was measured using techniques according to Standard Methods for the Examination of Water and Wastewater [29].

Water samples at the UNR were determined by atomic absorption spectroscopy (Arolab MK II, Buenos Aires, Argentina), by direct aspiration into acetylene-oxygen flame and absorbance at 424.0 nm. Calcium concentration was determined by comparison with a standard solutions of calcium (1–100 mg/L) processed simultaneously and in the same way, with a relative error of 0.5% and a relative standard deviation of 5.2%.

Calcium concentration of water samples at the UNLP was determined according to the titration method with ethylenediaminetetraacetic acid (EDTA). Water samples were diluted in a sodium hydroxide solution to produce a pH of 12–13 at which magnesium largely precipitates as the hydroxide. EDTA 0.01 M was added as titrant reagent until the Eriochrome Blue Black R indicated all calcium has been complexed. This methodology has the capacity to determine calcium content from 2 mg/L of CaCO_3_ with a relative error of 1.9% and a relative standard deviation of 9.2%.

Data from local authorities represent the calcium content of water at the distribution point whereas water samples measured at the laboratories represent calcium content at the drinking point. When possible samples from the same location source and analysed by the same methodology were aggregated and the mean and standard deviation was reported.

We also reported the calcium content described in the nutritional label of bottled waters commercially available in the Argentina. The period of data collection was between the years 2010 and 2018.

The contribution of water from different sources to calcium recommendations was made taking into account one litre of water per day and a calcium recommendation for adults of 1000 mg per day [30].

### Results

**Fig. 1:**
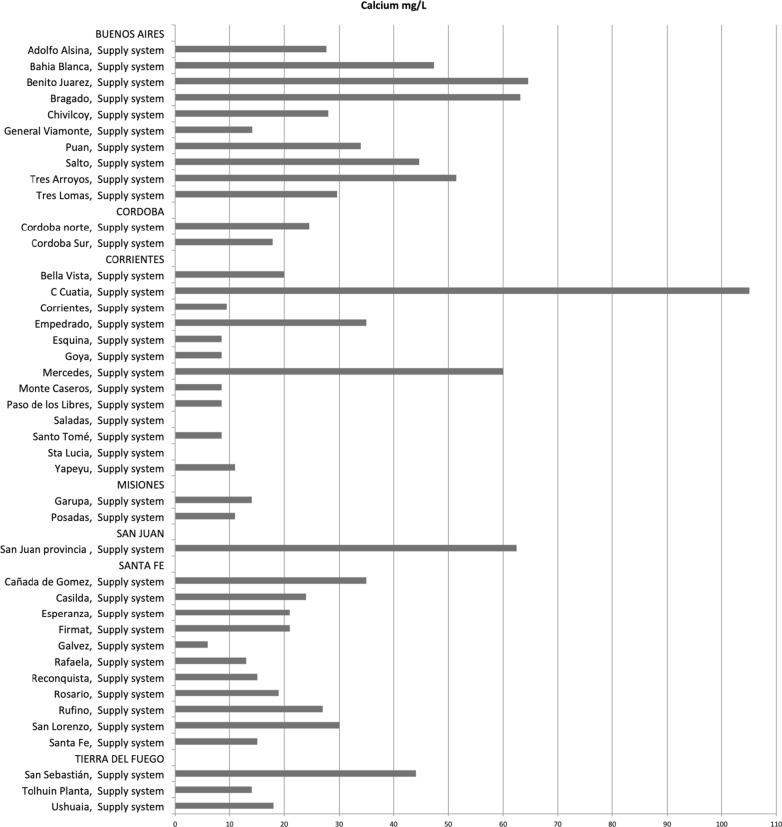
Calcium concentration (mg/L) from water samples from Water Local Authorities

We received information of water calcium concentrations from seven provincial water authorities that were classified as from supply systems: Water Control Agency of Buenos Aires (OCABA), Aguas Santafesinas Sociedad Anónima, Aguas Cordobesas, Federación Misionera de Cooperativas de Agua Potable, Aguas de Corrientes Sociedad Anónima, Obras Sanitarias San Juan Sociedad del Estado and Obras Sanitarias de Tierra del Fuego that provided data from 42 municipalities (Fig. 1).

**Fig. 2:**
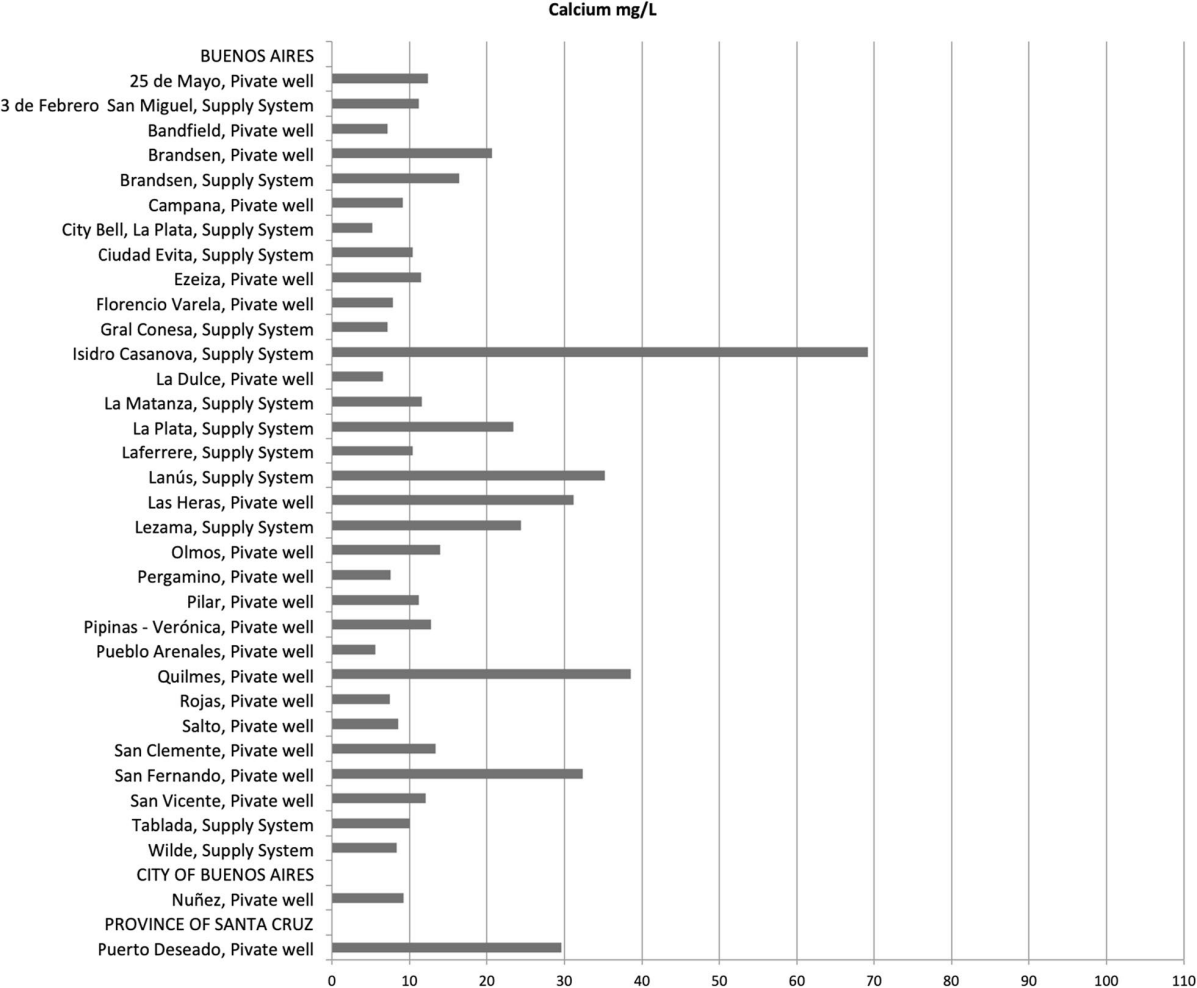
Calcium concentration (mg/L) from water samples remitted to the laboratory of UNLP

**Fig. 3:**
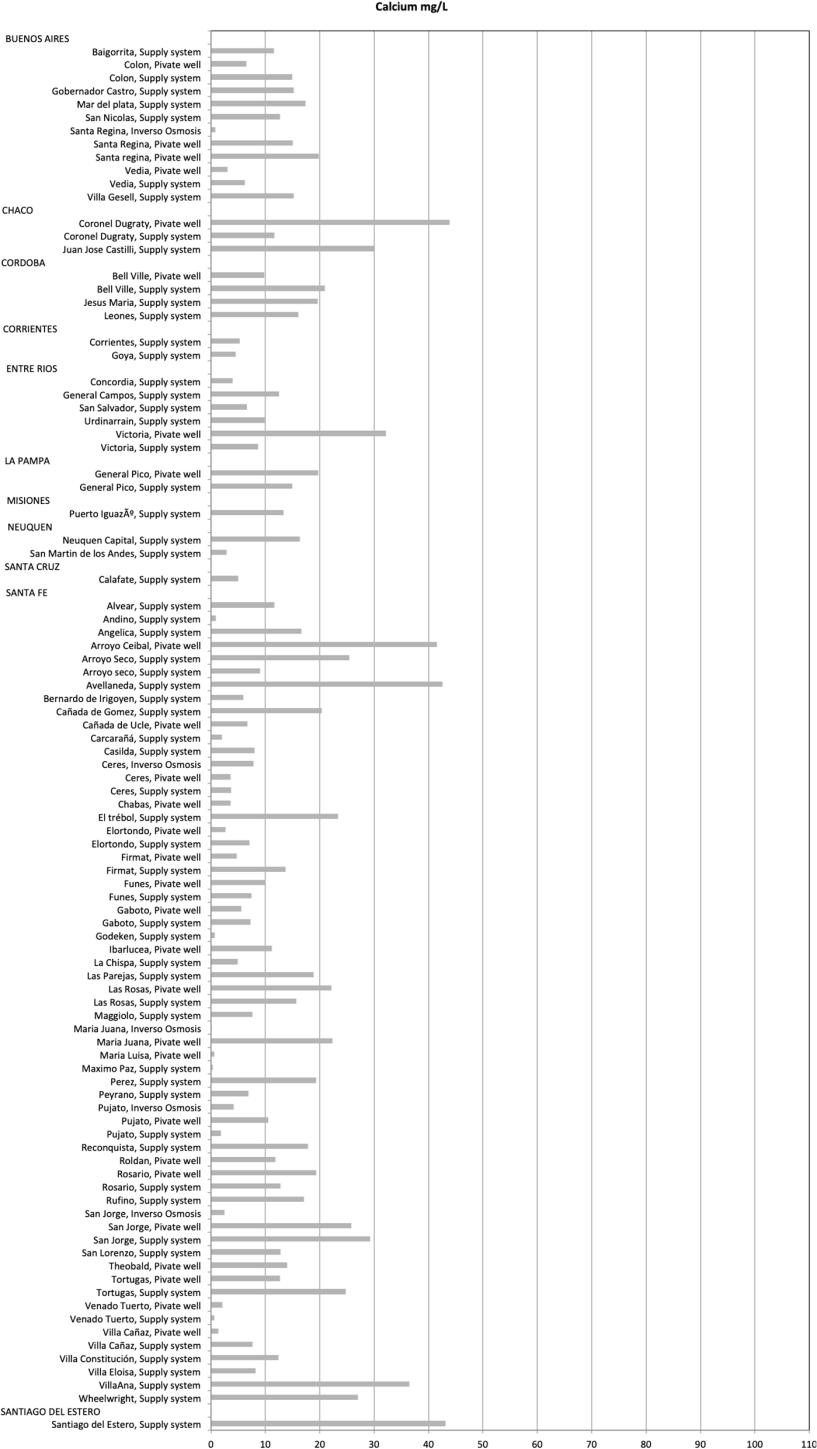
Calcium concentration (mg/L) from water samples remitted to UNR

We also had data from 184 samples remitted at the UNR from 72 municipalities of eleven provinces and 75 samples remitted at the UNLP from 34 municipalities of three provinces (Figs. 2, 3). Of the 184 samples remitted at the UNR 143 were from supply systems and 41 from private wells whereas of the 75 samples remitted at the UNLP, 14 were from supply systems and 61 from private wells.

Calcium concentrations of water provided by local authorities ranged from 6 to 105 mg/L, we were not able to average these values as in some cases data provided was already aggregated (Fig. 1, Additional file 1: TableS1). Mean calcium content of the water samples remitted at the laboratories was 15.8 (SD ± 13.2) mg/L ranging from 5.2 to 69.2 mg/L in UNLP and 13.1 (SD ± 10.0) mg/L in UNR ranging from 0.0 to 43.9 mg/L (Additional file 1: TableS2, S3 and S4). Water samples from supply system remitted at the UNLP had a mean calcium value of 19.0 (SD ± 16.8) whereas those remitted at the UNR had a mean calcium value of 12.9 (SD ± 8.8). Water samples from private wells remitted at the UNLP had a mean calcium value of 15.0 (SD ± 12.3) whereas those remitted at the UNR had a mean calcium value of 13.9 (SD ± 13.4) (Additional file 1: TableS4).

There were only six samples of water from local authorities that had calcium levels above 50 mg/L, three from the province of Buenos Aires (Benito Juarez, Bragado and Tres Arroyos), two from Corrientes (Curuzú Cuatiá and Mercedes) and one from San Juan (Fig. 1, Additional file 1: TableS1). Only one sample remitted at the UNLP had calcium levels above 50 mg/L whereas none of the samples remitted at UNR had levels above 50 mg/L (Figs. 2, 3, Additional file 1: TableS2, S3).

We found nine brands of still bottled water. The calcium values reported in the nutritional labels ranged from 11.5 to 80 mg/L. Only two labels reported calcium concentrations above 50 mg/L and none above 100 mg/L (Additional file 1: Table S5).

Taking into account a dietary recommendation of 1000 mg of calcium a day, the mean calcium concentrations found in the water samples remitted at the UNR and UNLP would represent from 1.3 to 1.9% of the recommended calcium daily values per litre of water consumed. Calcium concentrations reported by local authorities could provide between 0.6 and 10.5% of the recommended calcium daily values per litre of water consumed.

On the other hand, the amount of calcium found in bottled waters could provide between 1.2 and 8% of recommended daily values per litre of water consumed (Additional file 1: TableS5).

### Discussion

This article describes the calcium content of tap and commercially available drinking waters from Argentina. Data were obtained from local authorities representing calcium levels at the distribution point, from samples remitted and analysed in two public laboratories and from data of nutritional labels of commercially available bottled waters representing calcium levels at the drinking point. With a few exceptions, most tap or commercially available bottled waters had calcium levels well below 50 mg/L. The samples analysed at the laboratories had a mean calcium level of 15.8 (SD ± 13.2) mg/L for UNLP and 13.1 (SD ± 10.0) mg/L for UNR with similar calcium values between those from supply systems and those from private wells. The calcium content in water presented in this study shows high variability. Although samples are all from drinking water, water sources and treatment received were different and this can explain the variability. Even this variability, most samples had very little calcium concentration and the contribution to dietary intake seems to be marginal. The intake of a litre of tap water from samples analysed in our study would provide in average between 1.3 and 1.9% of the recommended daily calcium values for an adult, whereas the average calcium content of water provided by local authorities would provide an average of 2.8% and that from bottled water between 1.2 to 8.0% of the recommended daily calcium values for an adult.

An article describing mineral content of waters from the United States shows an average of 34 mg/L (SD ± 21) from tap waters of surface sources (n = 36), of 52 mg/L (SD ± 24) from tap water of ground sources and of 100 mg/L (SD ± 125) from mineral waters [6]. Information from Spain shows an average calcium concentration of public drinking waters of 38.9 (± 32.4) mg/L and for mineral water was 39.6 mg/L [31]. The calcium content of tap water and commercially available mineral bottled waters we present seem to be lower than those reported in the United States and Spain.

Water consumption is regularly omitted in dietary intake surveys and not all chemical composition tables report calcium content of water. The information reported in this article could be used in dietary surveys to estimate the calcium provided from drinking water in Argentina.

Due to the good bioavailability of calcium in water, rich calcium waters would be a good option to increase calcium intake. One review reports that water bioavailability was 23.8% with a 248 mg calcium load, however with lower loads bioavailability reached 47.5% [30] As water is usually drank thorough the day, the calcium load is spread and this can optimise calcium absorption.

There is evidence from epidemiological studies of an inverse relationship between water hardness and hypertension and also cardiovascular mortality [16–18]. In this way water hardness, which is determined by minerals in water, mainly calcium and magnesium, could improve health [19].

Taking into account the low calcium intake in many parts of the world and its consequences on health, regulations to improve calcium content of drinking water could help increasing calcium intake without changing dietary habits and consequently improve the health.

### Conclusion

Calcium concentrations found in tap and commercially available bottled waters in Argentina are negligible to significantly improve the calcium intake of the population. In view of the health benefits of adequate calcium intake, the low calcium intake in several regions of the world, increasing calcium of tap and commercially available bottled waters could be an alternative to universally and equitable improve calcium intake. Strategies to assess and test the feasibility to increase calcium content of water need to be developed.

## Limitations

- One of the limitations of this article is that the calcium content of water presented is not representative as it comes from 14 provinces of the 23 provinces in the country with no sampling methodology used or stated.
- Another limitation is that we do not have the methodology used to determine calcium values of data provided by local authorities nor the methodology used for measuring calcium concentration of commercially bottled waters.
- Although the methodologies used to determine calcium concentration of water by laboratories was different, both are accurate and recommended by the Standard Methods for the Examination of Water and Wastewater [29].

## Supplementary information

**Additional file1: TableS1.** Calcium content in tap water from cities in Argentina. Data provided by water local authorities.** TableS2**. Calcium content in tap water from analysed by the Laboratorio de Ingeniería Sanitaria National University of La Plata UNLP.** Table S3**. Calcium content in tap water from analysed by the Bone Biology Laboratory of the National University of Rosario (UNR).** Table S4**. Samples of water available. Sources UNLP (Universidad Nacional de la Plata); UNR (Universidad Nacional de Rosario).** Table S5**. Percentage of the daily calcium recommendations taking into account 1000 mg per day and 1, 1.5 and 2 litres of daily water intake of commercially available water bottles.

## Data Availability

All data generated or analysed during this study are included in this published article and its Additional file 1.

## Abbreviations

ENNyS: National Nutrition and Health Survey of Argentina
UNR: National University of Rosario
UNLP: National University of La Plata
EDTA: Ethylenediaminetetraacetic acid
OCABA: Water Control Agency of Buenos Aires
SD: Standard deviation
