# Supplementary material for: Contribution of calcium in drinking water from a South American country to dietary calcium intake
Source: BMC Res Notes. 2020 Oct 2;13:465. doi: 10.1186/s13104-020-05308-7 (PMC7532645; doi:10.1186/s13104-020-05308-7)
Supplement: Supplementary file 1 — Additional file1: TableS1. Calcium content in tap water from cities in Argentina. Data provided by water local authorities. TableS2. Calcium content in tap water from analysed by the Laboratorio de Ingeniería Sanitaria National University of La Plata UNLP. Table S3. Calcium content in tap water from analysed by the Bone Biology Laboratory of the National University of Rosario (UNR). Table S4. Samples of water available. Sources UNLP (Universidad Nacional de la Plata); UNR (Universidad Nacional de Rosario). Table S5. Percentage of the daily calcium recommendations taking into account 1000 mg per day and 1, 1.5 and 2 litres of daily water intake of commercially available water bottles. [file 13104_2020_5308_MOESM1_ESM.docx]

Additional file

| Additional file 1 **- Table S1: Calcium content in tap water from cities in Argentina. Data provided by water local authorities** | | | | |
| --- | --- | --- | --- | --- |
| **Province** | **Municipality** | **Source** | **n** | **Calcium (mg/L) Mean (SD)** |
| **Buenos Aires** | Adolfo Alsina | Supply system | 4 | 27.7 (16.4) |
|  | Bahia Blanca | Supply system | 18 | 47.3 (16.2) |
|  | Benito Juarez | Supply system | 11 | 64.6 (26.4) |
|  | Bragado | Supply system | 1 | 63.2 |
|  | Chivilcoy | Supply system | 3 | 28 (3.0) |
|  | General Viamonte | Supply system | 3 | 14.1 (5.5) |
|  | Puan | Supply system | 4 | 34 (2.0) |
|  | Salto | Supply system | 2 | 44.6 (32.0) |
|  | Tres Arroyos | Supply system | 13 | 51.5 (18.2) |
|  | Tres Lomas | Supply system | 5 | 29.6 (5.0) |
| **Córdoba** | Cordoba North | Supply system | 15 | 24.6 (3.3) |
|  | Cordoba South | Supply system | 14 | 17.8 (2.2) |
| **Corrientes** | Corrientes | Supply system | n/a | 9.5 |
|  | Santo Tomé | Supply system | n/a | 8.5 |
|  | Monte Caseros | Supply system | n/a | 8.5 |
|  | Paso de los Libres | Supply system | n/a | 8.5 |
|  | Yapeyú | Supply system | n/a | 9.5 |
|  | Mercedes | Supply system | n/a | 60 |
|  | Curuzú Cuatia | Supply system | n/a | 105 |
|  | Bella Vista | Supply system | n/a | 20 |
|  | Goya | Supply system | n/a | 8.5 |
|  | Esquina | Supply system | n/a | 8.5 |
| **Misiones** | Posadas | Supply system | 481 | 11 (0.8) |
|  | Garupa | Supply system | 141 | 14 (1.8) |
| **San Juan** | San Juan | Supply system | n/a | 62.5 |
| **Santa Fe** | Rosario | Supply system | n/a | 19 |
|  | Santa Fe | Supply system | n/a | 15 |
|  | Reconquista | Supply system | n/a | 15 |
|  | C de Gomez | Supply system | n/a | 35 |
|  | Casilda | Supply system | n/a | 24 |
|  | Esperanza | Supply system | n/a | 21 |
|  | Firmat | Supply system | n/a | 21 |
|  | Galvez | Supply system | n/a | 6 |
|  | Rafaela | Supply system | n/a | 13 |
|  | Rufino | Supply system | n/a | 27 |
|  | San Lorenzo | Supply system | n/a | 30 |
| **Tierra del Fuego** | San Sebastián | Supply system | n/a | 44 |
|  | Tolhuin | Supply system | n/a | 14 |
|  | Ushuaia | Supply system | n/a | 18 |
|  |  |  |  |  |

Additional file **- TableS2: Calcium content in tap water from analysed by the Laboratorio de Ingeniería Sanitaria**

**National University of La Plata UNLP**

|  |  |  |  | | Calcium (mg/L) |
| --- | --- | --- | --- | --- | --- |
| Province | Municipality | Source | n | Mean (SD) | |
| Buenos Aires | 25 de Mayo | Private well | 2 | 12.4 (1.1) | |
|  | 3 de Febrero. San Miguel | Supply system | 1 | 11.2 | |
|  | Bandfield | Private well | 1 | 7.2 | |
|  | Brandsen | Private well | 4 | 20.6 (6.0) | |
|  |  | Supply system | 1 | 16.4 | |
|  | Campana | Private well | 6 | 9.1 (2.8) | |
|  | City Bell | Supply system | 1 | 5.2 | |
|  | Ciudad Evita | Supply system | 1 | 10.4 | |
|  | Ezeiza | Private well | 7 | 11.5 (3.3) | |
|  | Florencio Varela | Private well | 4 | 7.9 (0.9) | |
|  | General Conesa | Supply system | 1 | 7.2 | |
|  | Isidro Casanova | Supply system | 1 | 69.2 | |
|  | La Dulce | Private well | 2 | 6.6 (0.3) | |
|  | La Matanza | Supply system | 1 | 11.6 | |
|  | La Plata | Supply system | 2 | 23.4 (2.0) | |
|  | Laferrere | Supply system | 1 | 10.4 | |
|  | Lanús | Supply system | 1 | 35.2 | |
|  | Las Heras | Private well | 4 | 31.2 (6.0) | |
|  | Lezama | Supply system | 1 | 24.4 | |
|  | Olmos | Private well | 1 | 14.0 | |
|  | Pergamino | Private well | 3 | 7.6 (9.4) | |
|  | Pilar | Private well | 1 | 11.2 | |
|  | Pipinas | Private well | 1 | 12.8 | |
|  | Pueblo Arenales | Private well | 1 | 5.6 | |
|  | Quilmes | Private well | 4 | 38.5 (22.7) | |
|  | Rojas | Private well | 4 | 7.5 (2.2) | |
|  | Salto | Private well | 5 | 8.6 (2.2) | |
|  | San Clemente | Private well | 2 | 13.4 (2.5) | |
|  | San Fernando | Private well | 1 | 32.4 | |
|  | San Vicente | Private well | 4 | 12.1 (8.1) | |
|  | Tablada | Supply system | 1 | 10.0 | |
|  | Wilde | Supply system | 1 | 8.4 | |
| Buenos Aires City | Nuñez (Gral Paz) | Private well | 1 | 9.2 | |
| Santa Cruz | Puerto Deseado | Private well | 3 | 29.6 (21.5) | |

Additional file1: **- Table S3: Calcium content in tap water from analysed by the Bone Biology Laboratory of the National University of Rosario (UNR)**

|  |  |  | Calcium (mg/L) | |
| --- | --- | --- | --- | --- |
| Province | Municipality | Source | n | Mean (SD) |
| Buenos Aires | Baigorrita | Supply system | 1 | 11.6 |
|  | Colón | Private well | 1 | 6.5 |
|  |  | Supply system | 3 | 14.9 (6.1) |
|  | Gobernador Castro | Supply system | 1 | 15.2 |
|  | Mar del Plata | Supply system | 1 | 17.4 |
|  | San Nicolas | Supply system | 5 | 12.7 (2.4) |
|  | Santa Regina | Supply system | 1 | 0.8 |
|  |  | Private well | 4 | 18.6 (21.8) |
|  | Vedia | Private well | 1 | 3 |
|  |  | Supply system | 1 | 6.2 |
|  | Villa Gesell | Supply system | 1 | 15.2 |
| Chaco | Coronel Dugraty | Private well | 2 | 43.9 (18.4) |
|  |  | Supply system | 1 | 11.7 |
|  | Juan Jose Castilli | Supply system | 1 | 30 |
| Cordoba | Bell Ville | Private well | 1 | 9.8 |
|  |  | Supply system | 3 | 20.9 (3.2) |
|  | Jesus Maria | Supply system | 1 | 19.6 |
|  | Leones | Supply system | 1 | 16.1 |
|  | Villa Carlos Paz | Supply system | 2 | 0.0 |
| Corrientes | Corrientes | Supply system | 1 | 5.3 |
|  | Goya | Supply system | 1 | 4.5 |
| Entre Rios | Concordia | Supply system | 1 | 4 |
|  | General Campos | Supply system | 1 | 12.5 |
|  | San Salvador | Supply system | 1 | 6.6 |
|  | Urdinarrain | Supply system | 3 | 9.9 (4.3) |
|  | Victoria | Private well | 1 | 32.2 |
|  |  | Supply system | 1 | 8.7 |
| La Pampa | General Pico | Private well | 3 | 19.7 (7.2) |
|  |  | Supply system | 39 | 14.9 (5.8) |
| Misiones | Puerto Iguazú | Supply system | 1 | 13.3 |
| Neuquén | Neuquén capital | Supply system | 1 | 16.3 |
|  | San Martin de los Andes | Supply system | 1 | 2.9 |
| Santa Cruz | Calafate | Supply system | 1 | 5.0 |
| Santa Fe | Alvear | Supply system | 1 | 11.7 |
|  | Andino | Supply system | 1 | 0.9 |
|  |  | Private well | 1 | 0.0 |
|  | Angelica | Supply system | 1 | 16.6 |
|  | Arroyo Ceibal | Private well | 1 | 10.8 |
|  | Arroyo seco | Supply system | 2 | 17.2 (11.6) |
|  | Avellaneda | Supply system | 1 | 42.6 |
|  | Bernardo de Irigoyen | Supply system | 1 | 5.9 |
|  | Cañada de Gomez | Supply system | 2 | 20.4 (0.2) |
|  | Cañada de Ucle | Private well | 4 | 6.7 (5.1) |
|  | Carcarañá | Supply system | 1 | 2.0 |
|  | Casilda | Supply system | 2 | 8.0 (5.9) |
|  | Ceres | Supply system | 2 | 5.8 (2.9) |
|  |  | Private well | 1 | 3.6 |
|  | Chabas | Private well | 1 | 3.6 |
|  | El trébol | Supply system | 3 | 23.4 (1.7) |
|  | Elortondo | Private well | 1 | 2.7 |
|  |  | Supply system | 1 | 7.1 |
|  | Firmat | Private well | 1 | 4.7 |
|  |  | Supply system | 3 | 13.7 (0.8) |
|  | Funes | Private well | 1 | 10 |
|  |  | Supply system | 1 | 7.4 |
|  | Gaboto | Private well | 1 | 5.6 |
|  |  | Supply system | 1 | 7.3 |
|  | Godeken | Supply system | 1 | 0.7 |
|  | Ibarlucea | Private well | 2 | 11.2 (1.5) |
|  | La Chispa | Supply system | 1 | 4.9 |
|  | Las Parejas | Supply system | 1 | 18.9 |
|  | Las Rosas | Private well | 1 | 22.1 |
|  |  | Supply system | 2 | 15.7(2.1) |
|  | Maggiolo | Supply system | 1 | 7.6 |
|  | Maria Juana | Supply system | 1 | 0.1 |
|  |  | Private well | 1 | 22.3 |
|  | Maria Luisa | Supply system | 1 | 0.0 |
|  |  | Private well | 2 | 0.3 (0.4) |
|  | Maria Susana | Supply system | 1 | 0.0 |
|  | Maximo Paz | Supply system | 2 | 0.1 (0.2) |
|  | Perez | Supply system | 3 | 19.3 (20.7) |
|  | Peyrano | Supply system | 1 | 6.9 |
|  | Pujato | Supply system | 2 | 3.0 (1.7) |
|  |  | Private well | 1 | 10.5 |
|  | Reconquista | Supply system | 1 | 17.8 |
|  | Roldan | Private well | 1 | 11.8 |
|  | Rosario | Private well | 2 | 19.3 (1.0) |
|  |  | Supply system | 9 | 11.4 (5.5) |
|  | Rufino | Supply system | 2 | 17.1 (0.2) |
|  | San Jorge | Supply system | 2 | 15.9 (19) |
|  |  | Private well | 1 | 25.8 |
|  | San Lorenzo | Supply system | 2 | 12.8 (14.7) |
|  | Theobald | Private well | 1 | 14.0 |
|  | Tortugas | Private well | 2 | 12.7 (2.5) |
|  |  | Supply system | 1 | 24.8 |
|  | Venado Tuerto | Private well | 1 | 2.1 |
|  |  | Supply system | 2 | 0.6 (0.1) |
|  | Villa Cañaz | Private well | 1 | 1.4 |
|  |  | Supply system | 2 | 7.6 (6.4) |
|  | Villa Constitución | Supply system | 2 | 12.4 (1.7) |
|  | Villa Eloisa | Supply system | 1 | 8.2 |
|  | Villa Ana | Supply system | 1 | 36.5 |
|  | Wheelwright | Supply system | 2 | 13.5 (19.1) |
| Santiago del Estero | Santiago del Estero | Supply system | 1 | 43.1 |

Additional file 1: Table S4: Samples of water available. Sources UNLP (Universidad Nacional de la Plata); UNR (Universidad Nacional de Rosario).

| Additional file **1: Table S5:** Percentage of the daily calcium recommendations taking into account 1000 mg per day and 1, 1.5 and 2 litres of daily water intake of commercially available water bottles. | | | | |
| --- | --- | --- | --- | --- |
|  |  | 1000 |  |  |
|  |  |  | Water intake (litre) | |
| Brand | Calcium (mg/L) | 1 | 1.5 | 2 |
| Evian | 80.0 | 8.0 | 12.0 | 16.0 |
| Villavicencio | 43.7 | 4.4 | 6.6 | 8.7 |
| Glaciar | 40.0 | 4.0 | 6.0 | 8.0 |
| Eco de los Andes | 30.0 | 3.0 | 4.5 | 6.0 |
| Cellier | 25.0 | 2.5 | 3.8 | 5.0 |
| San Francisco | 18.0 | 1.8 | 2.7 | 3.6 |
| Villa del Sur | 19.0 | 1.9 | 2.9 | 3.8 |
| Nestlé Pureza Vital | 15.0 | 1.5 | 2.3 | 3.0 |
| Villamanaos | 11.5 | 1.2 | 1.7 | 2.3 |
